# Updating the list of flower-visiting bees, hoverflies and wasps in the central atolls of Maldives, with notes on land-use effects

**DOI:** 10.3897/BDJ.10.e85107

**Published:** 2022-06-14

**Authors:** Paolo Biella, Axel Ssymank, Andrea Galimberti, Paolo Galli, Michal Perlík, Fausto Ramazzotti, Alessia Rota, Nicola Tommasi

**Affiliations:** 1 ZooPlantLab, Department of Biotechnology and Biosciences, University of Milano-Bicocca, Milano, Italy ZooPlantLab, Department of Biotechnology and Biosciences, University of Milano-Bicocca Milano Italy; 2 Bundesamt für Naturschutz, Bonn, Germany Bundesamt für Naturschutz Bonn Germany; 3 Earth and Environmental Science Department, University of Milano-Bicocca, Milano, Italy Earth and Environmental Science Department, University of Milano-Bicocca Milano Italy; 4 MaRHE Center (Marine Research and High Education Center), Magoodhoo, Maldives MaRHE Center (Marine Research and High Education Center) Magoodhoo Maldives; 5 Faculty of Science, University of South Bohemia, Ceske Budejovice, Czech Republic Faculty of Science, University of South Bohemia Ceske Budejovice Czech Republic; 6 Institute of Entomology, Biology Centre of the Czech Academy of Sciences, Ceske Budejovice, Czech Republic Institute of Entomology, Biology Centre of the Czech Academy of Sciences Ceske Budejovice Czech Republic

**Keywords:** DNA barcoding, distribution in the oriental region, oceanic island biodiversity, land use effect on pollinators, flower visitor, animals of tropical islands, ecosystem service of pollination, anthropogenetic habitat disturbance, Syrphidae, Anthophila, Apoidea, Vespoidea

## Abstract

Maldives islands host a unique biodiversity, but their integrity is threatened by climate change and impacting land-uses (e.g. cemented or agricultural areas). As pollinators provide key services for the ecosystems and for the inhabitants, it is crucial to know which pollinators occur in the islands, to characterise their genetic identity and to understand which plants they visit and the size of the human impact. Given that no significant faunistic surveys of Hymenoptera have been published for the country in more than 100 years and that Syrphidae were only partly investigated, we sampled islands in the central part of the Maldives country (Faafu and Daahlu atolls) and hand-netted flower-visiting bees, wasps and hoverflies (Hymenoptera: Anthophila, Crabronidae, Sphecidae, Vespidae, Scoliidae and Diptera: Syrphidae). Overall, we found 21 species; 76.4% of the collected specimens were Anthophila (bees), 12.7% belonged to several families of wasps and 10.8% of individuals were Syrphidae. It seems that one third of species are new for the Maldives, based on the published literature. Human land-uses seem to shape the local pollinator fauna since the assemblages of bees, wasps and hoverflies from urbanised and agricultural islands differed from those in resort and natural ones. These pollinators visited 30 plant species in total, although some invasive plants hosted the highest number of flower visitor species. Biogeographically, this pollinating fauna is mostly shared with Sri Lanka and India. Genetically, the used marker hinted for a unique fauna in relation to the rest of the distribution ranges in most cases, although generally within the level of intraspecific genetic variation. This study significantly contributes to increasing the knowledge on the pollinator diversity and genetic identity in Maldives islands also considering the important implications for the islands' land-use and the role of invasive plants. This study will be pivotal for future pollination studies and biodiversity conservation efforts in the region.

## Introduction

Pollinators are fundamental for guaranteeing cross-pollination for many plant species, some of which have high direct or indirect importance to human societies (Potts et al. 2016). It is well recognised that pollination is amongst the key ecosystem services for crops and wild plants (Silva et al. 2021), a service delivered by means of intricate interactions between pollinators and plants (Biella et al. 2019a, Biella et al. 2019b). For instance, about 90% of wild plants are estimated to be pollinated by animals (Ollerton et al. 2011). Moreover, pollinators greatly contribute to global food security by supporting about 75% of food production worldwide (Klein et al. 2007, Tscharntke 2021), providing also access to vitamins and other nutritional components (Porto et al. 2021). Even on islands, the pollination services are important for agricultural production of the inhabiting societies (Picanço et al. 2017) and the study and protection of pollinators there is a priority.

Pollinators and the ecosystems on small islands are facing an uncertain future due to climate change, human alteration of landscapes and invasive species (Harter et al. 2015, Sánchez-Ortiz et al. 2020). In particular, several island countries have suffered from rapid growths of urbanised or agriculture areas, further increasing the uncertainties to the conservation of the often unique island flora and fauna (Fallati et al. 2017, Steibl and Laforsch 2019, Tommasi et al. 2022). These factors add to existing constraints, mainly the small geographic ranges and isolation (Spengler et al. 2011), competitive invasive species becoming abundant (Kaiser-Bunbury et al. 2017) and increasing impact of human practices altering the landscape (Bissessur et al. 2017). The result of this combination of factors leads to small islands often hosting fewer bee or wasp species (Spengler et al. 2011) and bee assemblages of different composition (Zanella et al. 1998), compared to the mainland. In addition, islands usually select an immigrant pollinator fauna adapted to long dispersal, as, for instance, bee species are of moderate size and so they can undertake longer flights or bees are associated with a wood-nesting strategy allowing passive transport over water (Poulsen and Rasmussen 2020). Therefore, all these natural and artificial processes can directly determine the community composition of pollinators inhabiting an island.

Maldives islands are being increasingly transformed by human activities and this usually implies a growing impact on natural ecosystems (Fallati et al. 2017, Tommasi et al. 2022). The impact usually affects and transforms the features of the habitats that, in natural condition, would appear as composed by a foreshore with occasional creeping sand-binder plants, a beach crest hosting mainly shrubs adapted to wind and salt spray and the inner island with its dense forest of trees and shrubs (Selvam 2007). The animals and plants of Maldives are peculiar because these island are located in a key biogeographical position, being relatively near the Indian subcontinent, but also relatively close to islands, such as Mauritius and Seychelles, notorious stepping stones of species from Africa (Anderson 2009, Warren et al. 2010). Maldives have been well surveyed in terms of vertebrates, marine animals and plants (Pichon and Benzoni 2007, Factor and Shafeeqa 2010, Kitchen-Wheeler et al. 2012, Anderson et al. 2016), but the terrestrial invertebrate fauna is less known. More recent, albeit preliminary, surveys on this fauna regarded the orthopteroids, spiders and other insects of economic importance (Kevan and Kevan 1995, Watson et al. 1995, Steibl et al. 2020). In fact, a detailed investigation of pollinator communities in the Maldives islands has not taken place after a pioneering study on bees and wasps at the end of the 19^th^ century (Cameron 1901). Additional, but scattered contributions for Hymenoptera are hidden in later taxonomic studies on single genera comprising the Oriental Region (e.g. Blüthgen 1925, Reyes 1991), but they usually lack detailed distribution information for Maldives. Conversely, other frequent flower visitors, such as Syrphidae, have seldom been investigated in this country and firstly reported there only in 1995 for biological control (Watson et al. 1995), according to the published literature. Given the lack of recent surveys, the fragmented information on the distributions and the existing human footprint, it is, therefore, a priority to characterise the pollinator fauna of this region.

In this study, we surveyed several islands located in two atolls in the central part of the Republic of Maldives, characterised by different land-uses and levels of naturality. Here, we intend to fill the existing gap in the knowledge of the local fauna of flower-visitors, which is a recognised first step for updating the regional faunistic list of species and it is the basis for future studies and measures of conservation (Biella and Galimberti 2020, Cornalba et al. 2020). The aims were: (a) to characterise the distribution and genetic identities of flower-visiting bees, wasps and hoverflies in these islands; (b) to investigate how island land-use by humans shapes the composition of the flower-visiting fauna, also in relation to the visited flora.

## Methods

### Sampled islands

**Figure 1.**
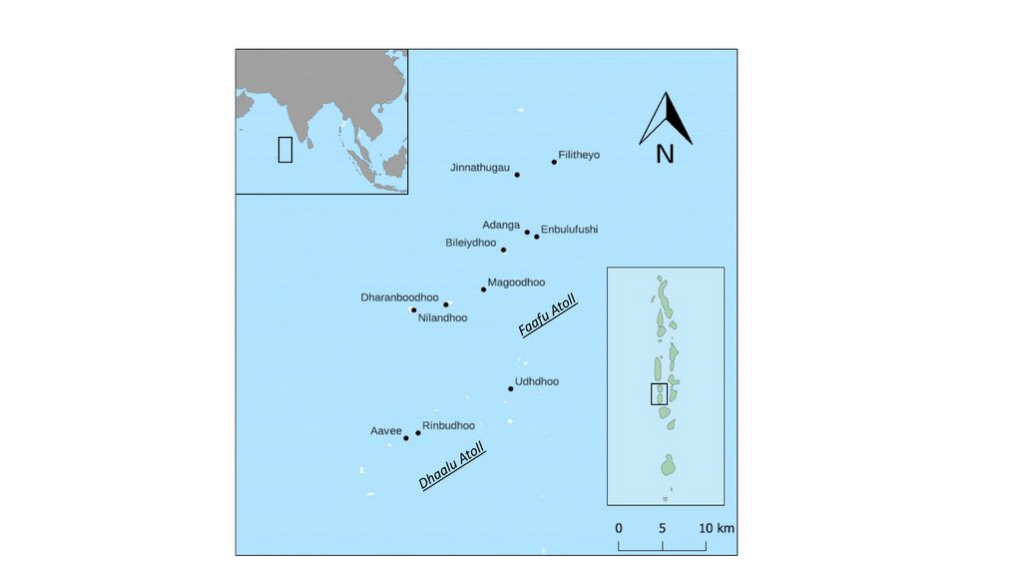
Sampled islands in the Faafu and Dhaalu atolls of Maldives.

The surveys took place on 11 islands of two adjacent atolls, Faafu and Daahlu, located in the central part of the Republic of Maldives (3.05537N 72.89129E, about 150 km south from the capital Male, Fig. 1). In Maldives, the climate is characterised by little seasonal variation around the mean temperature of 28°C and two monsoon periods (Bailey et al. 2015). The islands are geologically constituted by sands and gravels of reefs and they are small in terms of emerged surface (0.1-5 km^2^). They are either uninhabited, inhabited, dedicated to agriculture or resorts. Uninhabited islands are usually covered with dense forests occasionally mixed with shrubs and understorey plants. In islands dedicated to other uses, the typical coastal forest is intermixed with coastal strips of coconut plantations. The inner parts are occupied either by urban settlements or agricultural land or resorts. These land-uses can impact the vegetation, resulting in heterogeneous tree cover, scattered trees, cultivated fruit or ornamental plants intermixed with managed open areas. Non-native plant species cover almost 60% of the community composition of this archipelago (Sujanapal and Sankaran 2016). Lastly, island resorts are entirely dedicated to tourism, as they originated from previously uninhabited islands. These resorts vary greatly in terms of impact on the original natural environment, but usually they alternate patches of semi-natural, albeit managed forests, with gardens and touristic structures.

### Sampling

Between 16 October and 1 November 2019, entomological nets were used to collect flower-visiting insects along free transects crossing areas of homogeneous vegetation of about 50 x 50 m at each sampling location. Pollinators were collected during a fixed time of three hours per site between 9:00-16:00 h to facilitate comparison across sites, with uniform weather conditions across samplings. Specimens were stored individually in clean tubes with 70% ethanol solution. The plants where the flower visitors were captured were determined using Sujanapal and Sankaran (2016). The visited plants were also categorised as "native", "exotic non-invasive" and "invasive" following Sujanapal and Sankaran (2016) and Thomas (2011). Permissions relevant to undertake the research were obtained from the applicable governmental agencies. Specimens are stored in the collection of the ZooPlantLab of the University of Milano-Bicocca (https://www.gbif.org/grscicoll/institution/2936ff1c-6eb4-49eb-b728-8084ca2f9fa9). Overall, 17 sampling sites were included in the survey, distributed in different habitats of the islands (Table 1).

### Morphological identification and assessment of genetic similarities

All specimens were identified morphologically with standard keys and published taxonomical studies (Blüthgen 1925, Stuckenberg 1954, Reyes 1991, Lyneborg and Barkemeyer 2005). Detailed figures of reference specimens were also consulted for comparison from digitalised museum collections (e.g. Natural History Museum 2014). The following literature was used for the distribution of: Syrphidae (Watson et al. 1995, Lyneborg and Barkemeyer 2005, Gerlach 2009, Mitra et al. 2015, Thompson 2016, Ghorpadé 2019, Huo 2020, Ssymank et al. 2021), Crabronidae (van der Vecht 1949, Krombein 1984, Krombein and van der Vecht 1987), Scoliidae (Bradley and Betrem 1966, Krombein 1978), Sphecidae (Pham et al. 2015), Vespidae (Carpenter 1996, Nguyen et al. 2014, Pannure et al. 2016), Halictidae (Murao et al. 2020), Apidae (Maa 1938, Lieftinck 1957, Lieftinck 1964, Maa 1970, Reyes 1991, Shiokawa 2008), Megachilidae (Ascher et al. 2016), in addition to Cameron (1901). The resulting occurrence dataset is available on GBIF (Biella 2022).

Some specimens, selected randomly considering the distribution across islands, were processed to obtain standards full length DNA barcodes of the COI region (i.e. between 1 and 11 specimens per species). Genomic DNA was extracted from a leg following the laboratory protocols described in Cornalba et al. (2020). The Qiagen DNeasy Blood and Tissue Kit (Qiagen) were used according to manufacturer's instructions. Amplification of the 5’ end region of mitochondrial COI gene (658 bp) was carried out using primers LCO1490 and HCO2198 (Folmer et al. 1994) with the following PCR conditions: 94°C for 5 min, 5 cycles at 94°C for 60 s, 45°C for 90 s and 72°C for 90 s, 35 cycles at 94°C for 60 s, 54°C for 90 s and 72°C for 60 s and a final extension at 72°C for 7 min. Bidirectional sequencing of reverse strands only was performed at Eurofins Genomics using the same primer.

All sequences (along with specimen details) were submitted to the BOLD platform for statistical analyses under the project name ‘ZPLML Pollinator insects of Maldives’. Information on the genetic variation within-BIN (Barcode Index Number, Ratnasingham and Hebert 2013) were extracted, as well as the distance from the nearest neighbour BIN. In addition, the Batch ID Engine of BOLD was employed to obtain the percentage of distance between our Maldives sequences and all those with the same taxonomy, obtained by submitting our sequences to the identification tool of BOLD (in case of different taxonomy by same BIN, they were retained as possible cases of original misidentification in the genetic bank). This information was used as an indication of the average genetic distance of Maldives sequences from sequences from the rest of the distribution range.

Publicly available DNA barcode sequences, belonging to the same BINs found in Maldives, were downloaded from the BOLD Systems archive. Only the species for which more than 10 public sequences and from more than one country were available in the BIN were analysed (access date to BOLD Systems: 1 April 2022) by aligning nucleotide sequences from Maldives and BOLD systems (keeping sequences lengths above 500) and collapsing into unique haplotypes, using FaBox 1.5 (Villesen 2007). In order to investigate the frequency and geographic distribution of mtDNA haplotypes, Median Joining networks, encompassing the selected taxa (Suppl. material 1), were built using PopART 1.7 (Leigh and Bryant 2015).

### Environmental analysis

To better understand how the island main land-use associated with the composition of the pollinator assemblages, islands were categorised as natural, urban, agricultural and resort depending on the dominant land-use, after in-field evaluations with random transects across the islands. Assemblage composition in each site was tested with a Principal Coordinate Analysis (PCoA) with Bray-Curtis dissimilarity index. To test the significant differences amongst the main land-use types a Permutational Multivariate Analysis of Variance (PERMANOVA) with 999 permutations was performed. For these analyses, the R environment and the package *vegan* were used (Oksanen et al. 2018). The proportions of all sampled pollinators in the four main land-use types were represented with stacked bar-charts to easily compare the assemblage composition.

## Results

A total of 314 flower visitors was found, represented by 240 specimens of Anthophila (76.4%, Apidae, Megachilidae, Halictidae) belonging to eight species, 40 specimens of Sphecidae, Crabronidae, Vespidae and Scoliidae (12.7%) of eight species and 34 specimens of Syrphidae (10.8%) of five species. Frequently recorded bee species were: *Lasioglossumalbescens* (Smith, 1853), *Ceratinabinghami* Cockerell, 1908, *Braunsapispicitarsis* (Cameron, 1902), *Xylocopabryorum* (Fabricius, 1775) and *X.fenestrata* (Fabricius, 1798); amongst wasps: *Bembixborrei* Handlirsch, 1893 and *Campsomeriellacollaris* (Fabricius, 1775) were commonly captured; in hoverflies: *Syrittaproximata* Lyneborg & Barkemeyer, 2005, *Paragusserratus* (Fabricius, 1805) and *Eristalinuslaetus* (Wiedemann, 1830) were frequent (Table 1). Seven species we recorded seem to be new records for the country according to published literature (34% of the species, the distribution is shown in Table 2). Specifically, these new records were three Syrphidae and four Hymenoptera species. From a biogeographic point of view, this fauna from Faafu and Daahlu atolls show affinities with the known fauna of Sri Lanka (85% of taxa), India (95%) and South-East Asia (76%), while only three species are shared with Seychelles or Mauritius and two with Africa (Table 2).

**Figure 2.**
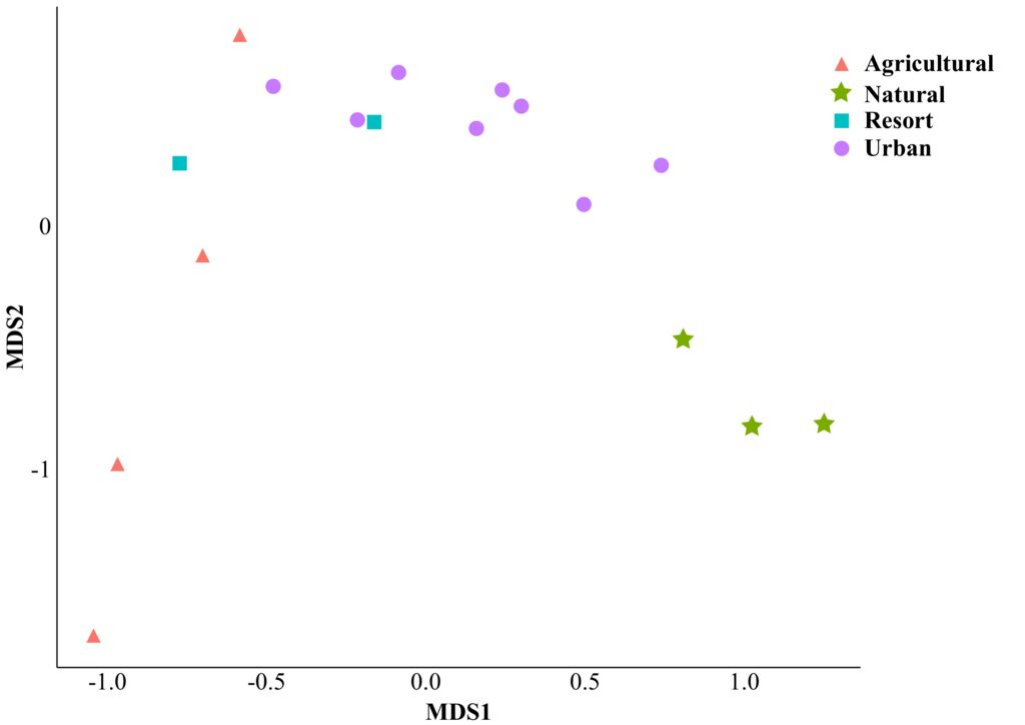
Ordination plot (PCoA) of flower visitor assemblages in sites depending on the main land-use types on the island (Agricultural, Natural, Resort, Urban).

**Figure 3.**
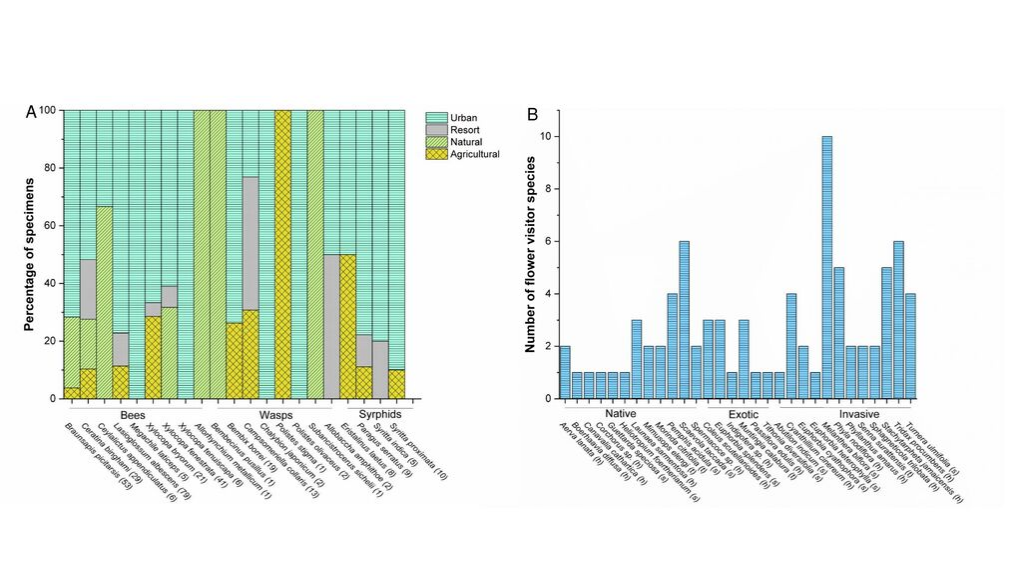
(A) Relative abundance of flower-visitors across different land-uses in the islands showing disharmonic community composition; on the x-axis, the species are grouped as “Bees”, “Wasps” and “Syrphids” and the number of individuals of each species is reported in brackets after species names; (B) Number of flower visitor species on each plant; on the x-axis, the plant species are grouped as native, exotic (non-invasive) and invasive.

When considering the main land-uses on the island, categorised as natural, urban, agricultural or resort islands (Fig. 2), the PCoA analysis of the pollinator assemblages indicated that the land-use type was a significant predictor (Fig. 2, the first two axes combined explained 45.3% of the variation). In particular, the urban landscape differed significantly from the resort, agricultural islands and natural ones, while agricultural sites were also significantly different from natural ones (Table 3). The pollinator species distributed unevenly in the main land-uses (Fig. 3A). These flower visitors interacted with a total of 30 plant species (Fig. 3B), the majority being herbs (50%) or shrubs (36.7%) and a few trees (13.3%). The plants hosting the higher number of visitor species were the invasive species *Melantherabiflora* (L.) Wild., *Tridaxprocumbens* L., *Phylanodiflora* (L.) Greene and *Stachytarphetajamaicensis* (L.) Vahl and the native *Scaevolataccada* (Gaertn.) Roxb.

**Figure 4.**
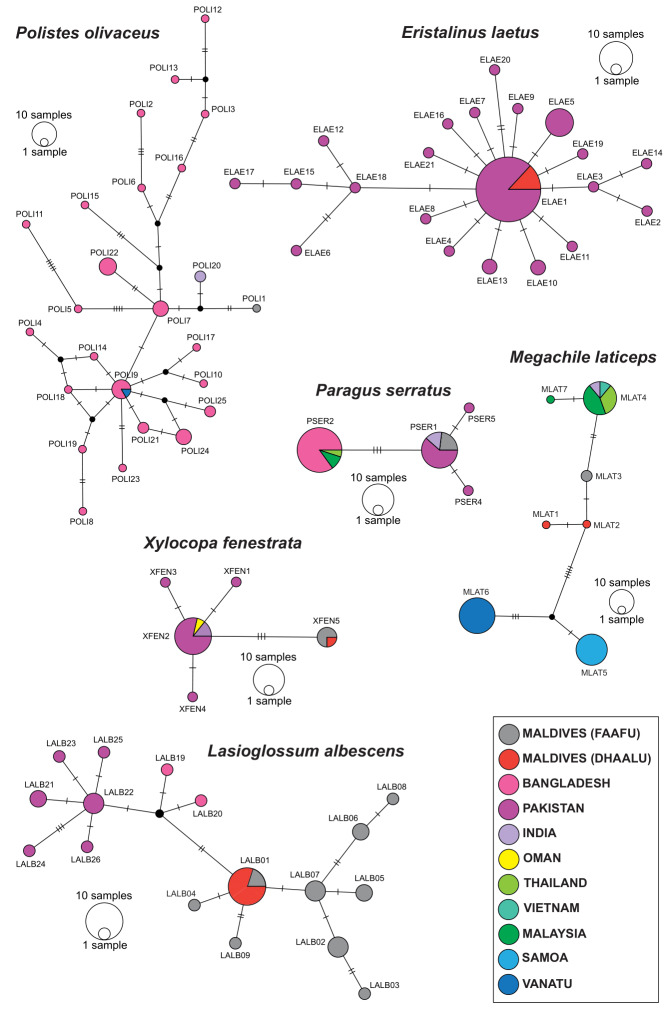
Median-Joining haplotype networks of some flower vistor species from the Maldives and other countries. Each circle represents a haplotype and its size is proportional to haplotype frequency (See Supplementary Table TS1). Colours indicate different countries and two Maldives atolls. Dashes represent substitutions, while black spots represent missing haplotypes.

Genetically, at the COI marker, the similarity of each species from Maldives to the other available sequences was considerable, with a mean across species of 98.85% (standard deviation 1.4, Table 4). In many cases, this distance was higher than the within-BIN average distances, but it was included in the within-BIN maximum distance. This is confirmed by the haplotype networks created on a subset of frequently barcoded species (Fig. 4). However, several cases had lower similarity scores and three cases yielded a particularly low mean similarity to other sequences: *Polistesstigma* (Fabricius, 1793) 94.07%, *Polistesolivaceus* (DeGeer, 1773) 97.57% and *Lasioglossumalbescens* (Smith, 1853) 97.87%. In four cases, the maximum within-BIN distances were particularly broad (i.e. above 2%): *Ceratinabinghami* Cockerell, 1908, *Eristalinuslaetus* (Wiedemann, 1830), *Lasioglossumalbescens* (Smith, 1853) and *Polistesolivaceus* (DeGeer, 1773).

## Discussion

In this study, we have contributed to updating the list of pollinating bees, wasps and hoverflies occurring in the Maldives and highlighted the relationships with different land-uses. After more than a century since the last published survey (Cameron 1901), here we contribute to the list of bees and wasps of the Maldives atolls. Moreover, we also updated the previous preliminary reports of the Syrphid fauna of these islands. Strikingly, as many as a third of the species we found had not previously been listed in this country according to literature. In spite of these novelties, the number of pollinator species in Maldives is low. However, it is likely that some additional species could be found if surveying more islands and for longer time, but studies on other subaerial arthropods still confirmed poorly diverse assemblages of spiders and orthopteroids (Kevan and Kevan 1995, Sunil 2012). A similar observation was made on Hymenoptera of other small islands, for instance, in an archipelago near Java (Spengler et al. 2011). This low species diversity is probably the result of geographic factors, such as small island size and isolation (Spengler et al. 2011).

Land-uses and the plant community might determine the pollinator fauna on small islands. We tested the importance of island main land-use on pollinator assemblages and recorded the flora visited there. Our results indicated an influence of human activities causing disharmonic communities across the archipelago, with some pollinator species becoming more frequent in some land-use types, but not in others. Sampling pollinators more intensively (e.g. for longer time and in more sites) or by using additional methods (e.g. pan-traps) could reveal further information on the effects of land-uses in these islands and even provide indications on habitat use by pollinators. Still, the standardised samplings we performed allowed us to compare pollinator groups in different anthropogenically affected islands. It seems particularly relevant that assemblages of urban and agricultural areas were significantly different from natural islands, clearly indicating the effects of human practices similarly to what is described for soil fauna in Maldives (Steibl et al. 2021). However, it is relevant to notice that human practices on islands can create novel niches in accordance with the intermediate disturbance hypothesis (Tommasi et al. 2022). In these islands, exotic invasive plants hosted the highest number of pollinators. This result reflects what was found in another study where island pollinators carried a higher amount of exotic plant species on their bodies (Tommasi et al. 2022). These findings agree with other studies indicating that invasive plants on islands could constitute a problem for pollination of the natives (Kaiser-Bunbury et al. 2017). Therefore, a careful evaluation of possible managment actions could prevent further impacts by land-use and by invasive plants.

The pollinator fauna in oceanic islands is the result of dispersal from neighbouring landmasses. For Maldives, most of the flower-visiting fauna is actually shared with India and Sri Lanka. This suggests that the main biogeographic route of colonisation is established with these lands, that are also the nearest ones. Similar patterns were also described for Maldives’ spiders (Sunil 2012) and it is perhaps a typical pattern of the subaerial animals there. However, a study reports observations of long dispersal of some large insects between Africa and India throughout the islands of the western Indian Ocean (Anderson 2009) and repeated exchanges over the geological time took place between India and Madagascar (Warren et al. 2010). Interestingly, after analysing a set of island vs. continent scenarios, a study found a higher proportion of wood nesting bees with increasing distance, as a result of dispersal by passive transportation over water (Poulsen and Rasmussen 2020). Active or, more likely, passive long dispersals were possible ways for Maldives colonisation by the pollinating fauna, but further studies are needed to confirm this hypothesis.

Colonisation from the neighbouring land-masses probably happened in ancient times because the genetic markers used in this study indicated differences from other parts of the distribution range. In pollinators such as bees, it is not uncommon to find genetic structuring that could be the result of historical events (Cornalba et al. 2020) and genetic divergence could even be amplified in island insects (De La Rúa et al. 1998, Magnacca and Danforth 2006). The emerging genetic differences of Maldives to the inland specimens is further highlighted for taxa with otherwise shallow genetic structuring across the surveyed range (i.e. the low mean distance within-BIN). In fact, only in a few cases, the high genetic distance was found in taxa with already a high within-BIN genetic amplitude. This suggests a peculiarity of Maldives pollinators under a genetic point of view. The fact that Maldives host a rather unique pollinator fauna adds further value to the need to increase the knowledge of pollinators inhabiting Maldives and how to possibly protect them.

In this study, after updating the distribution and check-list of the Maldives under a faunistic and genetic lens, we also clearly demonstrated that human practices affected the pollinator assemblages. Therefore, only by promoting suitable actions for island biodiversity conservation, the Maldives’ peculiar fauna could be preserved, for instance, with actions increasing the availability of feeding and nesting opportunities for various pollinators. Furthermore, increasing the awareness towards the local pollinator fauna will be very relevant given the importance of pollinators for food production, economy and human health (Potts et al. 2016).

## Supplementary Material

433390C1-109A-51A0-9F0A-AE7B7242BA8D 10.3897/BDJ.10.e85107.suppl1 Supplementary material 1 Supplementary Table - Details on haplotypes used in Figure 4. Data type Dataset of identifiers of genetic data. File: oo_670398.xlsx https://binary.pensoft.net/file/670398 Biella P, Ssymank A, Galimberti A, Galli P, Perlik M, Ramazzotti F, Rota A, Tommasi N.

## Figures and Tables

**Table 1.** Species identity, sampling locations and main land-use of the islands where bees, wasps and hoverflies were surveyed . “A”, “B” or “C” is indicated in case of several sites per island.

**Pollinator**	**Abundance**	**Flower species**
**Apidae**	**150**	
***Braunsapispicitarsis* (Cameron, 1902)**	**53**	*Cyanthilliumcinereum* (L.) H.Rob.; *Euphorbiacyathophora* Murray; *Launaeasarmentosa* (Willd.) Sch.Bip. ex Kuntze; *Melantherabiflora* (L.) Wild.; *Muntingiacalabura* L.; *Scaevolataccada* (Gaertn.) Roxb.; *Turneraulmifolia* L.
Adanga (Natural); 3.13897, 73.0083	3	
Bileiydhoo A (Urban); 3.11997, 72.9839	4	
Bileiydhoo B (Urban); 3.11535, 72.9864	5	
Dharanboodhoo A (Agricultural); 3.0613, 72.9243	1	
Enbulufushi (Natural); 3.13416, 73.0181	7	
Jinnathugau (Natural); 3.20051, 72.9979	3	
Magoodhoo A (Urban); 3.07754, 72.9633	3	
Magoodhoo B (Urban); 3.08023, 72.9658	2	
Magoodhoo C (Urban); 3.07667, 72.9600	12	
Nilandhoo A (Urban); 3.05537, 72.8912	3	
Nilandhoo B (Urban); 3.05675, 72.8866	1	
Rinbudhoo A (Urban); 2.92381, 72.8956	6	
Rinbudhoo B (Urban); 2.92525, 72.8942	3	
***Ceratinabinghami*** **Cockerell, 1908**	29	*Corchorus* sp.; *Indigofera* sp.; *Launaeasarmentosa* (Willd.) Sch.Bip. ex Kuntze; *Melantherabiflora* (L.) Wild.; *Pemphisacidula* J.R. Forst. e G. Forst.; *Scaevolataccada* (Gaertn.) Roxb.; *Stachytarphetajamaicensis* (L.) Vahl; *Tridaxprocumbens* L.
Aavee (Resort); 2.91822, 72.8832	1	
Bileiydhoo B (Urban); 3.11535, 72.9864	1	
Dharanboodhoo A (Agricultural); 3.0613, 72.9243	2	
Filitheyo (Resort); 2.91822, 72.8832	5	
Jinnathugau (Natural); 3.20051, 72.9979	5	
Magoodhoo A (Urban); 3.07754, 72.9633	1	
Magoodhoo B (Urban); 3.08023, 72.9658	2	
Nilandhoo A (Urban); 3.05537, 72.8912	1	
Rinbudhoo A (Urban); 2.92381, 72.8956	3	
Rinbudhoo B (Urban); 2.92525, 72.8942	7	
Udhdhoo (Agricultural); 2.97111, 72.9913	1	
***Xylocopabryorum (Fabricius, 1775)***	**21**	*Mimusopselengi* L.; *Morindacitrifolia* L.; *Passifloraedulis* Sims; *Pemphisacidula* J.R. Forst. e G. Forst.; *Scaevolataccada* (Gaertn.) Roxb.; *Sennasuratensis* (DC.) Irwin & Barneby
Bileiydhoo B (Urban); 3.11535, 72.9864	1	
Dharanboodhoo A (Agricultural); 3.0613, 72.9243	1	
Dharanboodhoo B (Agricultural); 3.06326, 72.9300	2	
Filitheyo (Resort); 2.91822, 72.8832	1	
Magoodhoo A (Urban); 3.07754, 72.9633	2	
Magoodhoo B (Urban); 3.08023, 72.9658	7	
Magoodhoo C (Urban); 3.07667, 72.9600	2	
Nilandhoo B (Urban); 3.05675, 72.8866	2	
Rinbudhoo B (Urban); 2.92525, 72.8942	2	
Udhdhoo (Agricultural); 2.97111, 72.9913	1	
***Xylocopafenestrata*** **(Fabricius, 1798)**	**41**	*Abutilonindium* (L.) Sweet; *Coleusscutellarioides* (L.) Benth.; *Melantherabiflora* (L.) Wild.; *Pemphisacidula* J.R. Forst. e G. Forst.; *Scaevolataccada* (Gaertn.) Roxb.; *Stachytarphetajamaicensis* (L.) Vahl; *Turneraulmifolia* L.
Aavee (Resort); 2.91822, 72.8832	3	
Adanga (Natural); 3.13897, 73.0083	8	
Bileiydhoo A (Urban); 3.11997, 72.9839	11	
Bileiydhoo B (Urban); 3.11535, 72.9864	2	
Enbulufushi (Natural); 3.13416, 73.0181	1	
Jinnathugau (Natural); 3.20051, 72.9979	4	
Magoodhoo A (Urban); 3.07754, 72.9633	2	
Magoodhoo C (Urban); 3.07667, 72.9600	1	
Nilandhoo A (Urban); 3.05537, 72.8912	5	
Rinbudhoo A (Urban); 2.92381, 72.8956	3	
Rinbudhoo B (Urban); 2.92525, 72.8942	1	
***Xylocopatenuiscapa* Westwood, 1840**	**6**	*Canavaliacathartica* Thouars; *Sennasuratensis* (DC.) Irwin & Barneby
Bileiydhoo B (Urban); 3.11535, 72.9864	2	
Magoodhoo C (Urban); 3.07667, 72.9600	3	
Rinbudhoo B (Urban); 2.92525, 72.8942	1	
**Crabronidae**	**20**	
***Bembecinuspusillus* (Handlirsch, 1892)**	**1**	*Guettardaspeciosa* L.
Enbulufushi (Natural); 3.13416, 73.0181	1	
***Bembixborrei* Handlirsch, 1893**	19	*Melantherabiflora* (L.) Wild.; *Phylanodiflora* (L.) Greene; *Sphagneticolatrilobata* (L.) Pruski; *Tridaxprocumbens* L.
Bileiydhoo A (Urban); 3.11997, 72.9839	3	
Bileiydhoo B (Urban); 3.11535, 72.9864	1	
Dharanboodhoo A (Agricultural); 3.0613, 72.9243	2	
Magoodhoo A (Urban); 3.07754, 72.9633	4	
Nilandhoo A (Urban); 3.05537, 72.8912	4	
Nilandhoo B (Urban); 3.05675, 72.8866	3	
Rinbudhoo A (Urban); 2.92381, 72.8956	2	
**Halictidae**	**85**	
***Ceylalictusappendiculatus* (Cameron, 1902)**	**6**	*Launaeasarmentosa* (Willd.) Sch.Bip. ex Kuntze; *Melantherabiflora* (L.) Wild.
Bileiydhoo B (Urban); 3.11535, 72.9864	1	
Enbulufushi (Natural); 3.13416, 73.0181	3	
Jinnathugau (Natural); 3.20051, 72.9979	1	
Magoodhoo C (Urban); 3.07667, 72.9600	1	
***Lasioglossumalbescens* (Smith, 1853)**	**79**	*Coleusscutellarioides* (L.) Benth.; *Cyanthilliumcinereum* (L.) H.Rob.; *Euphorbiacyathophora* Murray; *Euphorbiasplendens* Bojer ex Hook.; *Melantherabiflora* (L.) Wild.; *Mimusopselengi* L.; *Morindacitrifolia* L.; *Muntingiacalabura* L.; *Pemphisacidula* J.R. Forst. e G. Forst.; *Phylanodiflora* (L.) Greene; *Scaevolataccada* (Gaertn.) Roxb.; *Sphagneticolatrilobata* (L.) Pruski; *Stachytarphetajamaicensis* (L.) Vahl; *Tridaxprocumbens* L.; *Turneraulmifolia* L.
Aavee (Resort); 2.91822, 72.8832	6	
Bileiydhoo A (Urban); 3.11997, 72.9839	4	
Bileiydhoo B (Urban); 3.11535, 72.9864	8	
Dharanboodhoo A (Agricultural); 3.0613, 72.9243	6	
Filitheyo (Resort); 2.91822, 72.8832	3	
Magoodhoo A (Urban); 3.07754, 72.9633	10	
Magoodhoo B (Urban); 3.08023, 72.9658	12	
Magoodhoo C (Urban); 3.07667, 72.9600	8	
Nilandhoo A (Urban); 3.05537, 72.8912	2	
Nilandhoo B (Urban); 3.05675, 72.8866	3	
Rinbudhoo A (Urban); 2.92381, 72.8956	6	
Rinbudhoo B (Urban); 2.92525, 72.8942	11	
**Megachilidae**	**5**	
***Megachilelaticeps* Smith, 1853**	**5**	*Scaevolataccada* (Gaertn.) Roxb.; *Stachytarphetajamaicensis* (L.) Vahl
Magoodhoo B (Urban); 3.08023, 72.9658	2	
Rinbudhoo A (Urban); 2.92381, 72.8956	2	
Rinbudhoo B (Urban); 2.92525, 72.8942	1	
**Scoliidae**	**13**	
***Campsomeriellacollaris* (Fabricius, 1775)**	**13**	*Coleusscutellarioides* (L.) Benth.; *Cyanthilliumcinereum* (L.) H.Rob.; *Melantherabiflora* (L.) Wild.; *Muntingiacalabura* L.; *Stachytarphetajamaicensis* (L.) Vahl; *Tithoniadiversifolia* (Hemsl.) A.Gray; *Tridaxprocumbens* L.
Aavee (Resort); 2.91822, 72.8832	5	
Dharanboodhoo A (Agricultural); 3.0613, 72.9243	1	
Filitheyo (Resort); 2.91822, 72.8832	1	
Magoodhoo A (Urban); 3.07754, 72.9633	1	
Nilandhoo A (Urban); 3.05537, 72.8912	2	
Udhdhoo (Agricultural); 2.97111, 72.9913	3	
**Sphecidae**	**2**	
***Chalybionjaponicum* (Gribodo, 1883)**	**2**	*Euphorbiasplendens* Bojer ex Hook.
Magoodhoo B (Urban); 3.08023, 72.9658	2	
**Syrphidae**	**34**	
***Allobacchaamphithoe* (Walker, 1849)**	**2**	*Euphorbiasplendens* Bojer ex Hook.
Filitheyo (Resort); 2.91822, 72.8832	1	
Magoodhoo B (Urban); 3.08023, 72.9658	1	
***Eristalinuslaetus* (Wiedemann, 1830)**	**8**	*Tridaxprocumbens* L.; *Turneraulmifolia* L.
Nilandhoo B (Urban); 3.05675, 72.8866	1	
Rinbudhoo A (Urban); 2.92381, 72.8956	2	
Rinbudhoo B (Urban); 2.92525, 72.8942	2	
Udhdhoo (Agricultural); 2.97111, 72.9913	3	
***Paragusserratus* (Fabricius, 1805)**	**9**	*Boerhaaviadiffusa* L.; *Phylanodiflora* (L.) Greene; *Phyllanthusamarus* Schumach. & Thonn.; *Spermacoce* sp.
Bileiydhoo A (Urban); 3.11997, 72.9839	2	
Bileiydhoo B (Urban); 3.11535, 72.9864	1	
Dharanboodhoo A (Agricultural); 3.0613, 72.9243	1	
Filitheyo (Resort); 2.91822, 72.8832	1	
Magoodhoo A (Urban); 3.07754, 72.9633	1	
Magoodhoo C (Urban); 3.07667, 72.9600	1	
Nilandhoo A (Urban); 3.05537, 72.8912	2	
***Syrittaindica* (Wiedemann, 1824)**	**5**	*Aervalanata* (L.) A. L. Juss. ex Schultes; *Cyanthilliumcinereum* (L.) H.Rob.; *Melantherabiflora* (L.) Wild.; *Phylanodiflora* (L.) Greene
Filitheyo (Resort); 2.91822, 72.8832	1	
Magoodhoo A (Urban); 3.07754, 72.9633	2	
Magoodhoo C (Urban); 3.07667, 72.9600	1	
Rinbudhoo A (Urban); 2.92381, 72.8956	1	
***Syrittaproximata* Lyneborg & Barkemeyer, 2005**	**10**	*Aervalanata* (L.) A. L. Juss. ex Schultes; *Melantherabiflora* (L.) Wild.; *Phylanodiflora* (L.) Greene; *Phyllanthusamarus* Schumach. & Thonn.; *Spermacoce* sp.; *Tridaxprocumbens* L.
Bileiydhoo A (Urban); 3.11997, 72.9839	2	
Bileiydhoo B (Urban); 3.11535, 72.9864	1	
Dharanboodhoo A (Agricultural); 3.0613, 72.9243	1	
Magoodhoo A (Urban); 3.07754, 72.9633	2	
Magoodhoo C (Urban); 3.07667, 72.9600	2	
Nilandhoo A (Urban); 3.05537, 72.8912	1	
Rinbudhoo B (Urban); 2.92525, 72.8942	1	
**Vespidae**	**5**	
***Allorhynchiummetallicum* (de Saussure, 1853)**	**1**	*Melantherabiflora* (L.) Wild.
Enbulufushi (Natural); 3.13416, 73.0181	1	
***Polistesolivaceus* (DeGeer, 1773)**	**2**	*Euphorbiaheterophylla* L.
Bileiydhoo A (Urban); 3.11997, 72.9839	1	
Nilandhoo A (Urban); 3.05537, 72.8912	1	
***Polistesstigma* (Fabricius, 1793)**	**1**	
Udhdhoo (Agricultural); 2.97111, 72.9913	1	
***Subancistrocerussichelii* (de Saussure, 1856)**	**1**	*Heliotropiumfoertherianum* (Blanco) Mabb.
Jinnathugau (Natural); 3.20051, 72.9979	1	
**Total**	**314**	

**Table 2.** Biogeography of Maldives pollinators, with details on their distribution, based on literature. Species presence is indicated by “y”, its absence is indicated by “n”. See methods for the literature used. The species records new to Maldives are highlighted in bold.

**Species** **Order: Family**	**Previously in Maldives before this study**	**Indian subcontinent**	**Sri Lanka**	**S-E Asia**	**Seychelles; Mauritius**	**Africa**
*Braunsapispicitarsis* (Cameron, 1902) Hymenoptera: Apidae	y	y	y	n	n	n
*Ceratinabinghami* Cockerell, 1908 Hymenoptera: Apidae	y	y	y	n	n	n
*Xylocopabryorum* (Fabricius, 1775) Hymenoptera: Apidae	y	y	y	y	n	n
Xylocopafenestrata (Fabricius, 1798) Hymenoptera: Apidae	y	y	y	n	n	n
*Xylocopatenuiscapa* (Smith, 1853) Hymenoptera: Apidae	y	y	y	y	n	n
*Ceylalictusappendiculatus* (Cameron, 1902) Hymenoptera: Halictidae	y	n	y	n	n	n
***Lasioglossumalbescens* (Smith, 1853)** Hymenoptera: Halictidae	n	y	y	y	n	n
*Megachilelaticeps* (Smith, 1853) Hymenoptera: Megachilidae	y	y	n	y	y	n
*Campsomeriellacollaris* (Fabricius, 1775) Hymenoptera: Scoliidae	y	y	y	y	n	n
***Bembecinuspusillus* (Handlirsch, 1892)** Hymenoptera: Crabronidae	n	y	y	y	n	n
*Bembixborrei* (Handlirsch, 1893) Hymenoptera: Crabronidae	y	y	y	y	n	n
***Chalybionjaponicum* (Gribodo, 1883)** Hymenoptera: Sphecidae	n	y	n	y	n	y
*Allorhynchiummetallicum* (de Saussure, 1853) Hymenoptera: Vespidae	y	y	y	y	n	n
*Polistesolivaceus* (DeGeer, 1773) Hymenoptera: Vespidae	y	y	y	y	y	y
*Polistesstigma* (Fabricius, 1793) Hymenoptera: Vespidae	y	y	y	y	n	n
***Subancistrocerussichelii* (de Saussure, 1856)** Hymenoptera: Vespidae	n	y	y	y	y	n
*Allobacchaamphitoe* (Walker, 1849) Diptera: Syrphidae	y	y	y	y	n	n
***Eristalinuslaetus* (Wiedemann, 1830)** Diptera: Syrphidae	n	y	y	y	n	n
*Paragusserratus* (Fabricius, 1805) Diptera: Syrphidae	y	y	n	y	n	n
***Syrittaindica* (Wiedemann, 1824)** Diptera: Syrphidae	n	y	y	y	n	n
***Syrittaproximata* Lyneborg & Barkemeyer, 2005** Diptera: Syrphidae	n	y	y	n	n	n
**Number of species present**:	**14**	**20**	**18**	**16**	**3**	**2**

**Table 3.** Comparison of pollinator assemblages amongst the main land-use categories, based on sampling locations, tested with PERMANOVA. Statistics of the full model are given on the left part, while pairwise comparisons amongst land-uses are reported on the right side. Significant cases are reported in bold.

**Pseudo F value**	**P value of full model**	**P value from pairwise comparison**				
3.2332	0.001		Resort	Uninhabited	Urban	Agricultural
		Resort		0.1	**0.045**	0.342
		Uninhabited			**0.006**	**0.029**
		Urban				**0.003**
		Agricultural				

**Table 4.** Genetic distances at the COI marker, including BIN statistics, from the BOLD systems v.4 analysis tool. The “Mean distance to available sequences” refers to all sequences from the same taxonomical identity as those sequenced for Maldives. All values indicate percentages. “NA” is for cases sequenced exclusively from Maldives.

**Species** **Order: Family**	**Mean distance to available sequences**	**Average distance within-BIN**	**Maximum distance within-BIN**	**Distance to the nearest neighbour BIN**	**BIN identification number of Maldives specimens**
*Braunsapispicitarsis* (Cameron, 1902) Hymenoptera: Apidae	0.06	0.16	0.99	3.8	BOLD:ADT3151
*Ceratinabinghami* Cockerell, 1908 Hymenoptera: Apidae	0.46	0.13	2.46	3.16	BOLD:AAF1368
*Xylocopabryorum* (Fabricius, 1775) Hymenoptera: Apidae	0.51	0.27	0.54	2.35	BOLD:AEN0237
Xylocopafenestrata (Fabricius, 1798) Hymenoptera: Apidae	0.53	0.19	0.75	3.43	BOLD:AAE4670
*Xylocopatenuiscapa* (Smith, 1853) Hymenoptera: Apidae	0.09	0.14	0.48	3.92	BOLD:AEH7568
*Ceylalictusappendiculatus* (Cameron, 1902) Hymenoptera: Halictidae	NA	0	0	20.03	BOLD:AEM8210
***Lasioglossumalbescens* (Smith, 1853)** Hymenoptera: Halictidae	2.13	0.95	2.33	3.52	BOLD:AAN4354
*Megachilelaticeps* (Smith, 1853) Hymenoptera: Megachilidae	1.11	0.62	1.28	11.22	BOLD:AAK7030
*Campsomeriellacollaris* (Fabricius, 1775) Hymenoptera: Scoliidae	1.74	0.51	1.16	13.5	BOLD:AAZ8560
***Bembecinuspusillus* (Handlirsch, 1892)** Hymenoptera: Crabronidae	0.95	0.05	0.17	10.23	BOLD:AAQ3062
*Bembixborrei* (Handlirsch, 1893) Hymenoptera: Crabronidae	0.87	0.82	1.46	3.96	BOLD:AAV1401
***Chalybionjaponicum* (Gribodo, 1883)** Hymenoptera: Sphecidae	0.08	0.06	0.19	2.15	BOLD:ACL4327
*Allorhynchiummetallicum* (de Saussure, 1853) Hymenoptera: Vespidae	1.36	1.35	1.35	9.08	BOLD:ADR7001
*Polistesolivaceus* (DeGeer, 1773) Hymenoptera: Vespidae	2.41	0.76	2.25	2.16	BOLD:ADQ7771
*Polistesstigma* (Fabricius, 1793) Hymenoptera: Vespidae	5.93	0.82	0.82	6.05	BOLD:AEJ7215
***Subancistrocerussichelii* (de Saussure, 1856)** Hymenoptera: Vespidae	NA	0	0	15.03	BOLD:AEM6108
*Allobacchaamphitoe* (Walker, 1849) Diptera: Syrphidae	1.56	1.48	1.96	9.41	BOLD:ACY7012
***Eristalinuslaetus* (Wiedemann, 1830)** Diptera: Syrphidae	0.19	0.27	3.63	3.47	BOLD:AAU6733
*Paragusserratus* (Fabricius, 1805) Diptera: Syrphidae	0.44	0.21	1.69	1.81	BOLD:AAG4649
***Syrittaindica* (Wiedemann, 1824)** Diptera: Syrphidae	0.16	0.27	0.83	2.83	BOLD:AEN3709
***Syrittaproximata* Lyneborg & Barkemeyer, 2005** Diptera: Syrphidae	NA	0.68	1.5	2.83	BOLD:ADD9743
